# Embryonal prostatic rhabdomyosarcomas disguised presentation in an adolescent male: a case report

**DOI:** 10.4076/1757-1626-2-7546

**Published:** 2009-08-26

**Authors:** Nishith K Singh, Malatesha Gangappa, Vineet Gupta, Anant Mohan

**Affiliations:** 1 Department of Internal Medicine, Southern Illinois University School of MedicineSpringfield, IL, 62794USA; 2 Department of Medicine, Berkshire Medical CenterPittsfield, MA, 01201USA; 3 Department of Medicine, UPMC Mercy HospitalPittsburgh, PA, 15219USA; 4 Department of Internal Medicine, All India Institute of Medical SciencesNew Delhi, 110029India

## Abstract

Primary rhabdomyosarcoma of the prostate is a malignancy primarily of pediatric age group with most cases seen during infancy and childhood. Majority present with urinary symptoms but the case we report here presented at 16 years of age and had unusually nonspecific and temporally protracted nature of symptoms prior to the presentation. This remarkable unusualness lead to a delay in presentation and contributed to an eventually unfavorable outcome.

## Introduction

Primary embryonal prostatic rhabdomyosarcoma (EPRM) is an uncommon tumor and most cases, as reported in the past, present early in age and with predominant lower urinary tract symptoms [1,2]. We report a case which is unusual for its conspicuous absence of urinary symptoms or lymphadenopathy in spite of the widespread disease at presentation. The relatively nonspecific constitutional and protracted complaints of fever and polyarthralgias masquerading like that of a systemic illness, lead to a delayed presentation and poor prognosis for the patient.

## Case presentation

**Figure 1.**
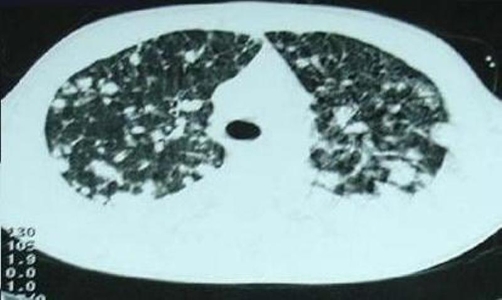
CECT chest (lung window) shows multiple varying nodules in both lungs.

**Figure 2.**
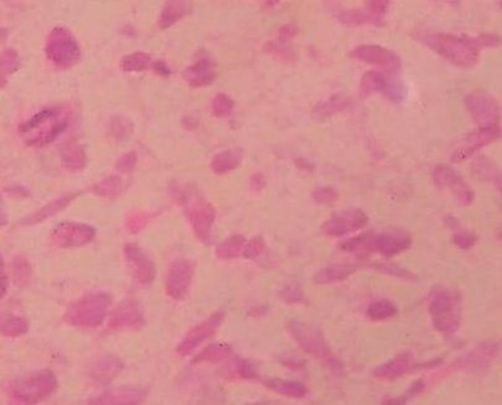
(H/E-400X) showing a malignant small round cell tumor with frequent mitosis and there is no evidence of any differentiation. Immunohistochemistry reveals focal cytoplasmic positivity for desmin and negativity for leukocyte common antigen, cytokeratin, chromogranin, MIC-2 and S-100 protein. The histological and histochemistry findings are compatible with undifferentiated rhabdomyosarcoma.

A 16-year-old Asian-Indian male student presented with progressively worsening dyspnea on exertion and new onset skin nodules over past one week. He has had polyarthralgias and low grade fever of two months duration. Past and personal history was unremarkable for any significant illness, allergy or high-risk behavior. Patient had received broad spectrum antibiotics prior to the admission for his constitutional symptoms without any clinical benefit. On examination the patient was febrile, pale, normotensive with sinus tachycardia, and had multiple non-tender subcutaneous nodules, two to four centimeters in size, predominantly on the scalp, face and upper trunk. He also had scattered crackles all over the chest, mild splenomegaly and non-tender prostate enlargement. Investigations showed normocytic normochromic anemia, hypercalcemia and hyperuricemia. Routine urinalysis was normal. No laboratory evidence of tuberculosis, immuno-compromised state or an autoimmune disorder was observed. Chest imaging (Figure 1) showed multiple bilateral nodular densities. Patient was hydrated, transfused blood products and put on an empirical broad spectrum antibiotic cover pending blood and urine culture reports which eventually turned out to be sterile. Computed tomography of abdomen unveiled an incidental, 6 × 5 × 5 cm homogenous soft tissue mass rising from prostrate. The prostate specific antigen (PSA) and prostatic acid phosphatase (PAP) were within normal limits. Trucut biopsy (of forehead nodule) was done; the microscopy and immuno-histochemistry suggested an embryonal rhabdomyosarcoma (Figure 2). Patient subsequently was found to have multiple osteolytic lesions in the skull, pelvis and proximal long bones. In view of a metastatic prostatic embryonal rhabdomyosarcoma the patient was started on vincristine, adriamycin and cyclophosphamide regimen. After a transient initial clinical response the patient succumbed to complications of widespread disease after 3 weeks. An autopsy could not be consented for by the patient’s relatives.

## Discussion

Embryonal prostatic rhabdomyosarcoma that occurs predominantly in male infants and children (median age of 5 years) is a rare and highly malignant tumour. Only sporadic cases have been reported in non-pediatric age group [1,2,3].

Characteristic features of EPRM published in literature include rapidly progressive obstructive urinary symptoms, smooth and firm enlargement of prostate on digital rectal examination, occasional suprapubic mass, regional lymph node spread, respiratory symptoms due to metastasis to lungs, osteoclastic bone metastases, and normal prostatic acid phosphates and prostate specific antigen levels [1,2,3]. The case presented here is atypical in terms of its inconspicuous presentation mimicking a systemic inflammatory response and in the authors’ best knowledge has not been reported before. Although primary site in prostate is a favorable prognostic sign for rhabdomyosarcomas, the presence of detectable metastases, tumor size > 5 centimeters and age > 10 years at presentation were the associated poor prognostic features in our patient which explains the unfortunate outcome [4]. Tumor debulking (excising ≥ 50 % of tumor) followed by chemotherapy has improved overall survival rates to 75% in EPRM patients, but our patient had distant detectable spread at the time of presentation which precluded any form of surgical intervention [5].

## Conclusion

Embryonal rhabdomyosarcoma of prostate in non-pediatric age group may have non specific presentation. It is prudent to have a high index of suspicion in diagnosing such cases early to avert grave prognosis.

## Abbreviations

EPRM: embryonal prostatic rhabdomyosarcoma
PAP: prostatic acid phosphatase
PSA: prostate specific antigen
